# Atypical development of white matter structural networks in children and adolescents with autism spectrum disorder: a graph theory study

**DOI:** 10.3389/fnins.2026.1844698

**Published:** 2026-08-25

**Authors:** Min Li, Kohei Kurita, Takashi Yamada, Yide Wang, Maya Izumoto, Yoshiko Iwatani, Ikuko Mohri, Qiulu Shou, Masatoshi Yamashita, Sayo Hamatani, Hideo Matsuzaki, Hidehiko Okazawa, Tokiko Yoshida, Hitomi Kitagawa, Tsuyoshi Sasaki, Koji Matsumoto, Tomomi Nagano, Yuko Isobe, Rio Kamashita, Yusuke Sudo, Eiji Shimizu, Ryo Kawasaki, Yoshiyuki Hirano, Yoshifumi Mizuno, Kuriko Kagitani-Shimono

**Affiliations:** 1 Division of Public Health, Department of Social Medicine, Graduate School of Medicine, The University of Osaka, Osaka, Japan; 2 United Graduate School of Child Development, The University of Osaka, Osaka, Japan; 3 Research Center for Child Mental Development, University of Fukui, Fukui, Japan; 4 Biomedical Imaging Research Center, University of Fukui, Fukui, Japan; 5 Cognitive Behavioral Therapy Center, Chiba University Hospital, Chiba, Japan; 6 Research Center for Child Mental Development, Chiba University, Chiba, Japan; 7 Department of Child Psychiatry, Chiba University Hospital, Chiba, Japan; 8 Department of Child Psychiatry, National Center Hospital, National Center of Neurology and Psychiatry (NCNP), Tokyo, Japan; 9 Department of Radiology, Chiba University Hospital, Chiba, Japan; 10 Institute for Advanced Academic Research, Chiba University, Chiba, Japan; 11 Department of Cognitive Behavioral Physiology, Graduate School of Medicine, Chiba University, Chiba, Japan; 12 Division of Clinical Neuroscience, Center for Forensic Mental Health, Chiba University, Chiba, Japan; 13 Department of Child and Adolescent Psychological Medicine, University of Fukui Hospital, Fukui, Japan

**Keywords:** autism spectrum disorder, developmental stages, diffusion tensor imaging, graph-theoretical analysis, social brain networks, white matter structural networks

## Abstract

**Background:**

Atypical brain connectivity is considered a key neurobiological feature underlying the heterogeneous clinical manifestations of autism spectrum disorder (ASD). However, findings on brain networks in ASD are inconsistent, likely owing to the effects of developmental factors. In addition, how large-scale brain networks in ASD differ across developmental stages remains unclear. We aimed to elucidate the atypical developmental patterns of white matter (WM) structural networks in children and adolescents with ASD using a graph-theoretical approach.

**Methods:**

Diffusion/T1-weighted brain imaging data were acquired from 69 individuals with ASD (age: 6–17 years) and 71 age- and sex-matched typically developing controls. Global and nodal topological properties of WM structural networks were computed, and 28 social-related regions were examined through subnetwork and nodal analyses. Case–control comparisons of global and nodal graph metrics were conducted separately for children and adolescents.

**Results:**

The children with ASD exhibited reduced integration of the whole-brain network, reflected by increased characteristic path length and decreased global efficiency. In contrast, the adolescents with ASD showed enhanced segregation within the social-brain subnetwork, indicated by increased clustering coefficient and local efficiency. Nodal analyses revealed reduced nodal efficiency across several social-related regions (e.g., the left inferior frontal gyrus, insula, amygdala, supramarginal gyrus, bilateral superior temporal poles) in children with ASD.

**Conclusion:**

Topological disorganization in the autistic brain network varies across developmental stages, shifting from reduced global integration in childhood to enhanced segregation of social-brain circuits in adolescence. Such atypical WM structural organization may underlie the persistent social cognitive deficits observed in ASD.

## 1 Introduction

Autism spectrum disorder (ASD) is a heterogeneous neurodevelopmental condition characterized by core deficits in social communication and interaction, as well as restricted and repetitive behaviors (American Psychiatric Association, 2013). Accumulating magnetic resonance imaging (MRI) evidence implicates atypical brain connectivity as a key neural mechanism underlying the heterogeneous symptoms of ASD (Hoppenbrouwers et al., 2014; Mohammad-Rezazadeh et al., 2016). However, the reported characteristics of brain connectivity in ASD remain inconsistent across studies (Ecker et al., 2015). Some studies have indicated widespread hypo-connectivity in both structural and functional connectivity (Jung et al., 2019; Just et al., 2012), whereas others have identified hyper-connectivity patterns in the autistic brain (Haghighat et al., 2021).

The developmental stage is likely a critical factor that contributes to these discrepant findings. Researchers have proposed that ASD manifests as a dynamic developmental syndrome, with distinct abnormal brain profiles exhibited at different developmental stages (Khundrakpam et al., 2017; Nunes et al., 2020). Uddin et al. (2013) demonstrated that the direction of connectivity alterations in ASD shows inverse patterns across developmental stages. However, most previous studies were focused on narrow or mixed age groups; thus, the moderating effects of developmental stages in the autistic brain remain unclear.

Understanding white matter (WM) connectivity across developmental stages is critical because myelination and axonal growth undergo rapid and extensive changes in early childhood, laying the structural foundation for the development of functional connectivity (Yu et al., 2020). Disruptions in WM microstructure can impair neural information transfer, thereby contributing to the cognitive and behavioral deficits observed in ASD (Travers et al., 2012). Diffusion tensor imaging (DTI), which quantifies the diffusion properties of water molecules in brain tissue, is an MRI technique widely used for assessing WM connectivity. DTI-derived metrics have shown good reliability in pediatric and multisite datasets (Kamagata et al., 2015; Merisaari et al., 2019); numerous DTI studies have reported widespread WM abnormalities in ASD (Andica et al., 2021; Lei et al., 2019). Our recent meta-analysis of 31 DTI studies demonstrated that individuals with ASD exhibit reduced integrity across multiple WM tracts (Li et al., 2022). Furthermore, our subsequent study showed that WM disruptions are closely associated with both language comprehension and symptom severity, with alterations being more pronounced in younger children than in adolescents (Li et al., 2024). Although WM abnormalities tend to attenuate with development, neural alterations and autistic manifestations rarely normalize completely. Human brain development progresses from local cortical neuronal connectivity to large-scale network organization, through the myelination of white matter fibers and pruning of connections (Ecker et al., 2015; Fishman et al., 2015). Therefore, we hypothesized that with the advancement in intrinsic brain development, connectivity abnormalities in ASD extend beyond alterations in specific WM tracts to large-scale network disturbances.

Graph theory is an analytical framework that conceptualizes complex network systems as graphs composed of nodes and edges. In brain-network analysis, cortical regions are defined as nodes, whereas the structural or functional connections between regions are represented as edges. This approach allows for quantification of the topological properties of brain networks using graph-derived metrics. Graph theory has been utilized in previous studies to examine the topological characteristics of the autistic brain; however, the findings remain inconsistent (Kim et al., 2021; Qin et al., 2018; Roine et al., 2015). As discussed above, the developmental stages may substantially contribute to discrepancies in brain connectivity alterations in ASD. However, the developmental dynamics of topological disorganization in the autistic brain were not sufficiently addressed in previous network studies. In addition, most existing studies were focused on whole-brain-network topology, whereas the social brain subnetwork, which is critically involved in social cognition and communication, has received comparatively limited attention. To our knowledge, no previous study was conducted using DTI-based graph-theoretical analysis to investigate the topological characteristics of the social brain subnetwork in ASD.

In the present study, we applied graph-theoretic analysis on relatively large DTI datasets to compare the topological organization of whole-brain and social-brain subnetworks between individuals with ASD and typically developing (TD) controls. In addition, we conducted subgroup analyses to investigate how atypical WM network topology varies across developmental stages. The subgroup analyses primarily focused on intrinsic developmental changes in structural brain networks, rather than on experience-dependent compensatory processes. We expect that our findings will contribute to a deeper understanding of the intrinsic brain-developmental mechanisms underlying network-level abnormalities in ASD.

## 2 Methods and materials

### 2.1 Participants

Participants with ASD (aged 6–17 years) were recruited through the Child Developmental MRI project, which involves the University of Osaka, the University of Fukui, and Chiba University (Yamashita et al., 2023). ASD was diagnosed by experienced pediatric clinicians or child psychiatrists based on the Diagnostic and Statistical Manual of Mental Disorders, Fifth Edition. TD controls were recruited from the local community. The controls had no history of developmental, neurological, or psychiatric disorders and had never received special education. Intelligence was assessed using the Wechsler Intelligence Scale for Children-Fourth or Fifth Edition. Autistic symptoms were measured using the Social Responsiveness Scale-Second Edition (SRS-2), a 65-item caregiver-report questionnaire that comprises five subscales: social awareness, social cognition, social communication, social motivation, and autistic mannerisms (Constantino, 2013). The inclusion criteria for both groups were as follows: (1) full-scale IQ (FSIQ) > 75; (2) no history of head injury or neurological illness; and (3) right-handedness as examined using the Edinburgh Handedness Inventory. To investigate developmental changes, the participants were stratified into children (6.0 < = age < 12.0 years) and adolescents (12.0 < = age < 17.0 years).

The study protocol was approved by the Institutional Review Board’s Ethics Committee at each university (Assurance No. K22213). All participants and their parents provided written informed consent after receiving a full explanation of this research.

### 2.2 MRI acquisition

Diffusion and T1-weighted images were acquired using 3-T MRI scanners at three institutions (Supplementary Table 1). High-resolution T1-weighted images were obtained using axial protocols established at each institution. Diffusion-weighted images (DWI) were acquired using a single-shot echo-planar imaging (EPI) sequence. All participants received standardized instructions before undergoing the procedure. We used cushions and foam pads to stabilize each participant’s head within the coil.

### 2.3 Data pre-processing and network construction

DICOM images were first converted to NIfTI format using MRIcroGL,^1^ and all scans were visually checked for artifacts using MANGO (v4.1; https://mangoviewer.com/mango.html).

Preprocessing and network construction were subsequently performed using PANDA (Cui et al., 2013), an automated pipeline toolbox that integrates the FMRIB Software Library and the Diffusion Toolkit. PANDA provides a standardized workflow that encompasses preprocessing, cortical parcellation, fiber tractography, and the generation of WM connectivity matrices. The default preprocessing steps include the following (1) estimating the brain mask from the b0 image to serve as an initial reference for further processing; (2) cropping non-brain areas in the raw images to reduce computational load; (3) correcting for eddy current distortions and head motion by registering the diffusion-weighted images to the b0 image; (4) fitting diffusion tensors using the ditfit command to compute FA maps.

**Figure 1:**
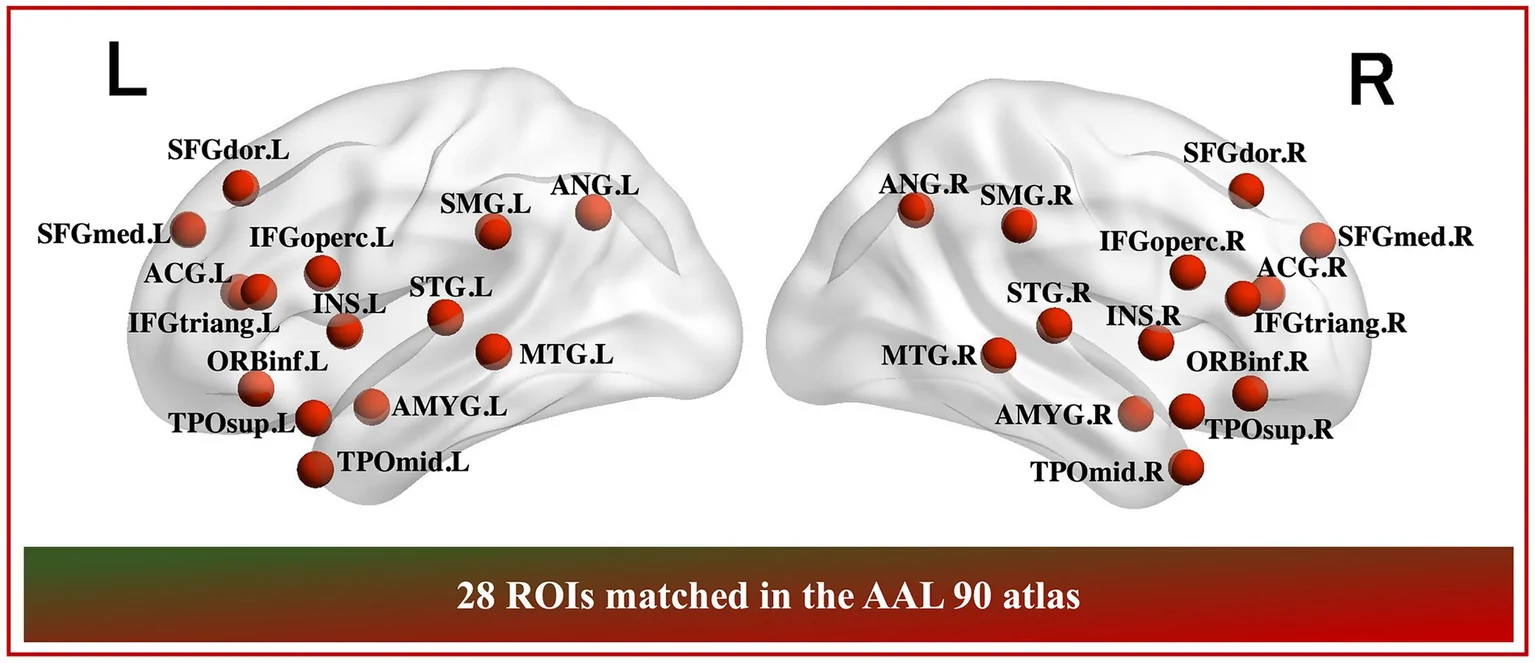
Twenty-eight ROIs in the social-brain subnetwork defined in this study. Regions of the social brain were defined based on information in the existing literature (Blakemore, 2008). These regions included the anterior cingulate cortex (ACC), medial prefrontal cortex (mPFC), amygdala, anterior insula, inferior frontal gyrus, adjacent temporo-parietal junction (TPJ), superior temporal sulcus (STS), and temporal poles. The regions were mapped onto the Automated Anatomical Labeling (AAL) atlas, yielding 28 corresponding ROIs (red dots) for subnetwork and nodal analyses. Detailed information on the anatomic partitions of the ROIs and their Montreal Neurological Institute (MNI) coordinates are listed in Table 1. L, Left; R, Right; ROI, region of interest.

Network construction involved defining the network nodes and edges. Network nodes were defined using the Automated Anatomical Labeling (AAL) atlas, which comprises 90 cerebral regions. As the AAL atlas is defined in standard space, a two-step registration procedure was applied to transform it into the DTI native space. FA images were first registered to T1 images and then to the ICBM152 template, with inverse transforms applied to warp the AAL atlas back to native space. Network edges were defined based on fiber bundles connecting pairs of cortical nodes. Whole-brain deterministic fiber tractography was performed in PANDA, terminating when FA < 0.2 or the turning angle > 30°. To reduce the risk of false-positive connections and to improve the robustness of the constructed WM networks, only connections present in at least 80% of participants were retained. Additionally, a structural connection was retained only when ≥3 streamlines connected two regions. Subsequently, a number-weighted WM connectivity matrix was generated for each participant for subsequent topological analyses. WM connectivity matrices were constructed for the whole brain (90 × 90) and the social brain subnetwork (28 × 28). Regions of the social brain subnetwork, including 28 regions of interest (Figure 1), were defined based on information in previous studies (Blakemore, 2008) and mapped to their corresponding labels in the AAL atlas (Table 1).

**Table 1:** Detailed information on the functional and anatomic partitions of the ROIs within the social brain subnetwork.

Regions of the social-brain subnetwork (Blakemore, 2008)	ROIs matched in the AAL 90 atlas	Abbreviation (labels)	*MNI*		
			*x*	*y*	*z*
Adjacent anterior cingulate cortex (ACC)	Anterior cingulate and paracingulate gyri	ACG L (31) ACG R (32)	−4.04 8.46	35.40 37.01	13.95 15.84
Medial prefrontal cortex (mPFC)	Superior frontal gyrus, medial	SFGmed L (23) SFGmed R (24)	−4.80 9.10	49.17 50.84	30.89 30.22
	Superior frontal gyrus, dorsolateral	SFGdor L (3) SFGdor R (4)	−18.45 21.90	34.81 31.12	42.20 43.82
Amygdala	Amygdala	AMYG L (41) AMYG R (42)	23.27 27.32	−0.67 0.64	−17.14 −17.50
Anterior insula (AI)	Insula	INS L (29) INS R (30)	−35.13 39.02	6.65 6.25	3.44 2.08
Inferior frontal gyrus (IFG)	Inferior frontal gyrus, opercular part	IFGoperc L (11) IFGoperc R (12)	−48.43 50.20	12.73 14.98	19.02 21.41
	Inferior frontal gyrus, triangular part	IFGtriang L (13) IFGtriang R (14)	−45.58 50.33	29.91 30.16	13.99 14.17
	Inferior frontal gyrus, orbital part	ORBinf L (15) ORBinf R (16)	−35.98 41.22	30.71 32.23	−12.11 −11.91
Adjacent temporo-parietal junction (TPJ)	Supramarginal gyrus	SMG L (63) SMG R (64)	−55.79 57.61	−33.64 −31.50	30.45 34.48
	Angular gyrus	ANG L (65) ANG R (66)	−44.14 45.51	−60.82 −59.98	35.59 38.63
Posterior superior temporal sulcus (pSTS)	Superior temporal gyrus	STG L (81) STG R (82)	−53.16 58.15	−20.68 −21.78	7.13 6.80
	Middle temporal gyrus	MTG L (85) MTG R (86)	−55.52 57.47	−33.80 −37.23	−2.20 −1.47
Temporal poles	Temporal pole: superior temporal gyrus	TPOsup L (83) TPOsup R (84)	−39.88 48.25	15.14 14.75	−20.18 −16.86
	Temporal pole: middle temporal gyrus	TPOmid L (87) TPOmid R (88)	−36.32 44.22	14.59 14.55	−34.08 −32.23

AAL, Automated Anatomical Labeling; ROIs, regions of interest; MNI, Montreal Neurological Institute; L, Left; R, Right.

### 2.4 Graph-theoretical analysis

Topological metrics were computed from the fiber-number-based connectivity matrix of each participant using the GRETNA toolbox, an open-source MATLAB package used for graph-theoretical analysis (Wang et al., 2015). The topological organization of the WM structural network was characterized using global and nodal graph measures.

Four global network metrics, including characteristic path length (L_p_), global efficiency (E_glob_), clustering coefficient (C_p_), and local efficiency (E_loc_), were estimated for the whole-brain network and the social brain subnetwork. L_p_ and E_glob_ represent network integration, reflecting the efficiency of information transfer across long-range brain regions. C_p_ and E_loc_ quantify network segregation, indicating the capacity for specialized processing supported by densely interconnected local networks. Nodal efficiency (E_nodal_) was computed to assess the regional contribution to network-wide information transfer. E_nodal_ quantifies the efficiency of information exchange between a given brain region and the rest of the network and is considered a comprehensive measure of the brain region’s capacity for global information integration (Sporns, 2018; Farahani et al., 2019). In addition, because E_nodal_ incorporates information from the entire brain network, it reportedly exhibits good robustness and test–retest reliability in brain-network studies (Termenon et al., 2016; Rubinov and Sporns, 2010). Therefore, we selected E_nodal_ as the primary nodal metric to evaluate alteration in the information integration of social brain regions in ASD. Nodal analyses were focused on the 14 social cognition-related regions per hemisphere described in Figure 1.

### 2.5 Statistical analysis

Demographic and clinical characteristics were compared between the ASD and TD groups using two-sample t-tests in JASP (v.0.17.3; https://jasp-stats.org). Graph metrics were then harmonized using a traveling-subject (TS) approach implemented in Python. The TS method is a novel harmonization technique that effectively mitigates measurement biases arising from differences in MRI scanners or imaging protocols across multisite datasets (Yamashita et al., 2019). We compared the TS and Combat correction methods in our recent study (Shou et al., 2025) and demonstrated that the TS method can address measurement bias more effectively, while preserving sampling specificity relative to Combat. Details of the TS datasets have been described in our previous work (Shou et al., 2025). Between-group comparisons of the harmonized graph metrics at global and nodal levels were performed using a general linear model, with sex and age included as covariates. Given that global network efficiency is closely associated with IQ level (Li et al., 2009; Suprano et al., 2020), we further conducted sensitivity analyses of global graph metrics by including FSIQ as an additional covariate. Homogeneity of variances was assessed using Levene’s test. For graph metrics that violated the assumption of equal variances, Welch’s t-test was additionally performed, and results were compared with those obtained using Student’s *t*-test. The group comparisons were conducted separately in the children and adolescent groups to investigate developmental changes in atypical topological organization in ASD.

Statistical analyses for graph metrics were performed using the metric comparison module of the GRETNA toolbox. Nodal comparisons were corrected using the false discovery rate (FDR) because of the large number of statistical tests. For the global comparison, primary analyses were based on uncorrected *p-values* (*p* < 0.05), given that only four prior measures were examined. In addition, FDR-corrected results were reported to enhance the robustness evaluation of the findings.

## 3 Results

### 3.1 Demographic and clinical characteristics

We recruited 71 individuals with ASD. Two datasets were removed from the analysis due to poor registration quality, leaving 69 individuals with ASD (mean age = 11.58 ± 2.92 years; 36 children and 33 adolescents) included in the final analyses. MRI datasets from 71 age- and sex-matched TD controls (mean age = 11.34 ± 3.11 years; 38 children and 33 adolescents) were included for analysis. The demographic and clinical characteristics of the participants are presented in Table 1. No significant between-group differences in age were observed (*p* > 0.05). The ASD group showed significantly lower FSIQ scores (*T* = −4.51, *p* < 0.001) and poorer performance in the four cognitive subscales (VCI: *T* = −4.01, *p* < 0.001; PRI: *T* = −2.20, *p* = 0.030; PSI: *T* = −3.68, *p* < 0.001; WMI: *T* = −2.99, *p* = 0.003) than the TD controls. The ASD group also exhibited markedly higher SRS-2 scores, indicating pronounced social deficits (all *p* < 0.001). The subgroup analysis of the adolescents showed non-significant case–control differences in FSIQ scores and most cognitive indices, except for PSI (*T* = −2.69, *p* = 0.009). Nevertheless, adolescents with ASD continued to exhibit significantly higher SRS-2 scores than TD controls (Table 2).

**Table 2:** Demographic and clinical characteristics of participants.

Demographic and clinical characteristics	ASD mean (SD)	TD mean (SD)	*p* value	T value
Participants (*n*)	69	71	-	-
Univ. of Osaka (*n*)	18	17	-	-
Univ. of Fukui (*n*)	44	41	-	-
Chiba Univ. *(n)*	7	13	-	-
Sex (*n.* male/female)	57/12	52/19	-	-
Age (*years*)	11.58 (2.92)	11.34 (3.11)	0.632	0.480
FSIQ	95.44 (12.90)	106.15 (13.31)	< 0.001^**^	−4.51
VCI	98.19 (15.43)	109.04 (13.94)	<0.001^**^	−4.01
PRI	98.65 (14.39)	104.69 (13.96)	0.030^*^	−2.20
PSI	93.90 (13.55)	103.51 (14.79)	<0.001^**^	−3.68
WMI	92.49 (12.77)	100.16 (15.17)	0.003^**^	−2.99
SRS-2				
Total scores	63.16 (22.91)	25.85 (14.20)	<0.001^**^	10.33
Mannerisms	68.22 (13.46)	47.09 (7.63)	< 0.001^**^	9.96
SCI	64.26 (11.25)	45.94 (7.65)	< 0.001^**^	9.80
Awareness	58.45 (10.65)	44.48 (9.37)	<0.001^**^	7.15
Cognition	65.35 (12.26)	46.65 (8.99)	<0.001^**^	8.95
Communication	63.80 (11.25)	45.67 (6.85)	<0.001^**^	10.04
Motivation	59.31 (12.62)	49.41 (8.27)	<0.001^**^	4.78
*Subgroup*				
Children group (*n*)	36	38	-	-
FSIQ	92.89 (12.53)	106.85 (11.37)	< 0.001^**^	−4.55
VCI	95.03 (13.33)	109.19 (12.14)	< 0.001^**^	−4.33
PRI	97.60 (15.23)	107.64 (12.88)	0.013^*^	−2.57
PSI	93.33 (14.31)	102.27 (13.75)	0.017^*^	−2.47
WMI	89.61 (9.84)	99.96 (14.21)	0.001^**^	−3.39
SRS-total	65.52 (10.09)	46.41 (7.12)	< 0.001^**^	7.69
Mannerisms	68.76 (11.43)	47.11 (7.32)	< 0.001^**^	7.97
SCI	63.91 (9.79)	46.48 (7.06)	< 0.001^**^	7.17
Awareness	59.52 (10.93)	46.33 (9.45)	<0.001^**^	4.48
Cognition	65.00 (10.88)	45.85 (8.13)	<0.001^**^	6.98
Communication	62.14 (9.10)	45.56 (6.35)	<0.001^**^	7.43
Motivation	60.42 (13.47)	51.30 (8.33)	<0.001^**^	2.89
Adolescent group (*n*)	33	33	-	
FSIQ	98.31 (12.89)	105.46 (15.13)	0.053	−1.98
VCI	101.97 (16.95)	108.89 (15.71)	0.108	−1.63
PRI	99.84 (13.53)	101.87 (14.65)	0.601	−0.53
PSI	94.53 (12.84)	104.80 (15.98)	0.009^**^	−2.69
WMI	95.72 (14.92)	100.36 (16.40)	0.269	−1.12
SRS-total	65.90 (12.88)	45.56 (8.11)	< 0.001^**^	7.04
Mannerisms	67.83 (14.90)	47.07 (8.08)	< 0.001^**^	6.44
SCI	64.50 (12.32)	45.41 (8.30)	< 0.001^**^	6.78
Awareness	57.70 (10.57)	42.63 (9.09)	<0.001^**^	5.74
Cognition	65.60 (13.32)	47.44 (9.85)	<0.001^**^	5.80
Communication	64.97 (12.56)	45.78 (7.44)	<0.001^**^	6.92
Motivation	58.53 (12.16)	47.52 (7.91)	<0.001^**^	4.01

ASD, autism spectrum disorder; TD, typically developing; SD, standard deviation; FSIQ, full scale intelligence quotient; VCI, verbal comprehension index; PRI, perceptual reasoning index; PSI, processing speed index; WMI, working memory index; SRS-2, social responsiveness scale, second- edition; SCI, social communication and interaction; *p < 0.05, **p < 0.01.

### 3.2 Comparative analyses of global topological metrics

**Figure 2:**
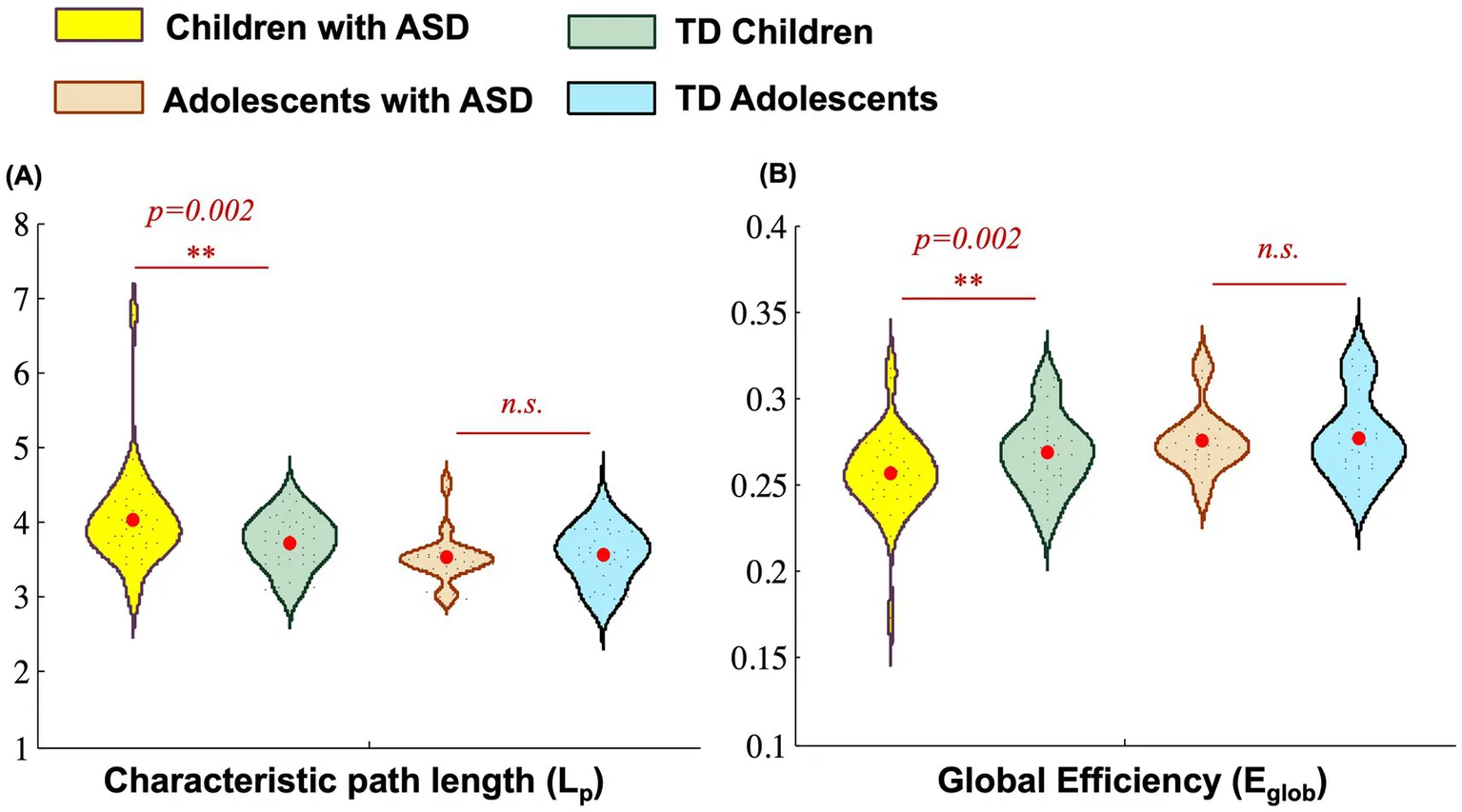
Between-group comparisons of global topological metrics within the whole-brain network. **(A)** Significantly increased L_p_ of the whole-brain network in the children with ASD (*p* = 0.002, *FDR-corrected*). **(B)** Significantly decreased E_glob_ of the whole-brain network in the children with ASD (*p* = 0.002, *FDR-corrected*). No statistically significant differences in L_p_ or E_glob_ were observed in the adolescent group. Light yellow represents children with ASD, light green denotes TD children, orange indicates adolescents with ASD, and cyan blue represents TD adolescents. ** Indicates a statistically significant difference between groups, *p* < 0.01, with FDR correction. ASD, autism spectrum disorder; TD, typically developing.

Regarding whole-brain global properties, children with ASD showed significantly higher L_p_ (*T* = 3.242, *p* = 0.002, *FDR-corrected*) and lower E_glob_ (*T* = −3.158, *p* = 0.002, *FDR-corrected*) than TD controls. No significant differences were observed in adolescents (Figures 2A,B). In addition, no significant alterations in E_loc_ and C_p_ at the whole-brain level were detected in either age group (Supplementary Figure 1). The group difference in L_p_ and E_glob_ in children remained significant after adjustment for FSIQ (L_p_: *T = 2.990, p = 0.004, FDR-corrected;* E_glob_*: T = −2.916, p = 0.005, FDR-corrected*).

**Figure 3:**
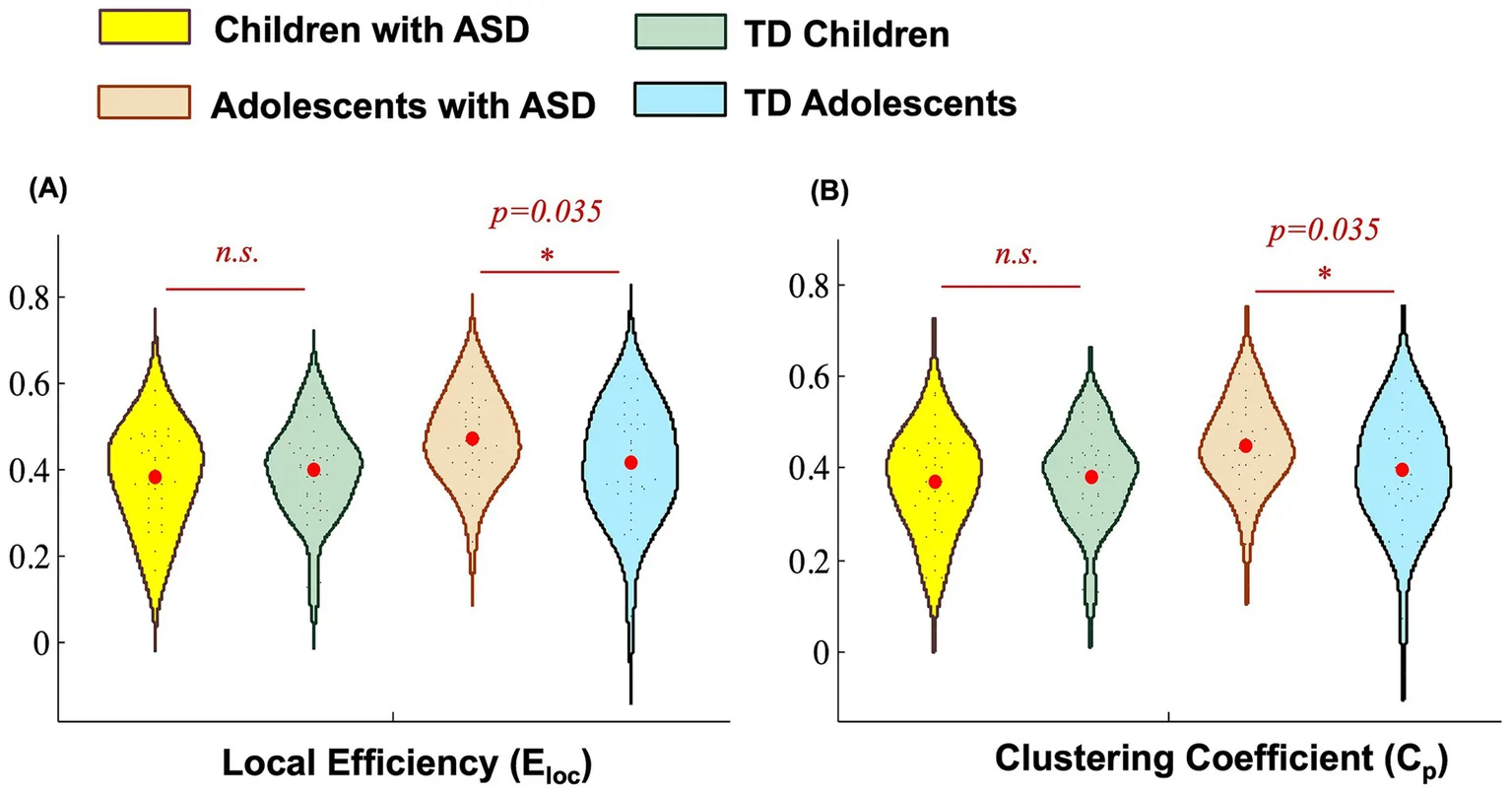
Between-group comparisons of global topological metrics within the social-brain subnetwork. **(A)** Significantly increased Eloc of the social-brain subnetwork in adolescents with ASD (*p* = 0.035, *uncorrected*). **(B)** Significantly increased Cp of the social-brain subnetwork in adolescents with ASD (*p* = 0.035, *uncorrected*). No statistically significant differences in Eloc or Cp were observed in the children group. Light yellow represents the children with ASD, light green denotes the TD children, orange indicates the adolescents with ASD, and cyan blue represents TD adolescents. * indicates a statistically significant difference between groups, *p* < 0.05, *uncorrected*. ASD, autism spectrum disorder; TD, typically developing.

Regarding the social-brain subnetwork, no significant differences in L_p_ or E_glob_ were observed in either age group (Supplementary Figure 2). At the subnetwork level, adolescents with ASD showed significantly higher E_loc_ (*T* = 1.938, *p* = 0.035, *uncorrected*) and C_p_ (*T* = 1.954, *p* = 0.035, *uncorrected*) than TD controls. However, no significant case–control differences in these metrics were observed in the children group (Figures 3A,B). After additionally adjusting for FSIQ, group differences in E_loc_ and C_p_ in adolescents were attenuated (*E_loc_: T = 1.965, p = 0.053, uncorrected; C_p_: T = 2.000, p = 0.0499, uncorrected*). Moreover, these subnetwork-level findings in adolescents did not survive FDR correction.

### 3.3 Comparative analyses of nodal topological metrics

**Figure 4:**
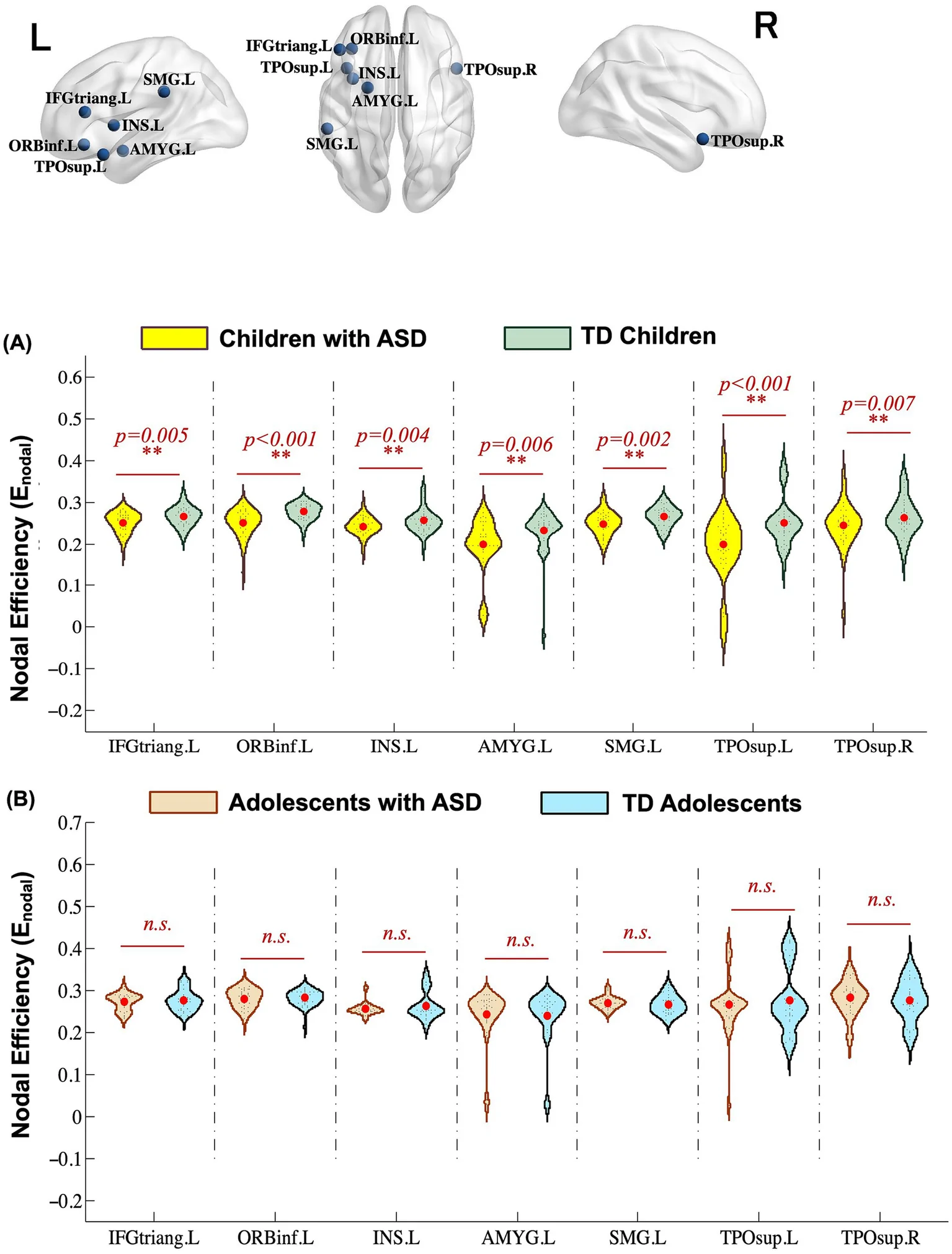
Between-group comparisons of nodal efficiency in ROIs involved in social cognition. **(A)** Case–control comparisons of nodal efficiency in the children group (light yellow represents children with ASD and light green denotes the TD children). Children with ASD showed decreased E_nodal_ in widespread social brain regions, including the triangular and orbital parts of the left IFG, left INS, AMYG, SMG, and the bilateral superior parts of the TPO (FDR correction, *p* < 0.05). **(B)** Case–control comparisons of nodal efficiency in the adolescent group (orange indicates the adolescents with ASD, and cyan blue represents the TD adolescents. No significant group differences were observed between the TD adolescents and those with ASD). ** indicates a statistically significant difference between groups, *p* < 0.01 with FDR correction. ASD, autism spectrum disorder; TD, typically developing; IFG, inferior frontal gyrus; ORBinf, orbital parts of inferior frontal gyrus; INS, insula; AMYG, amygdala; SMG, supramarginal gyrus; TPO, temporal poles; L, Left; R, Right.

At the local network level, nodal efficiency in the 28 regions implicated in social cognition was compared between groups. Children with ASD exhibited significantly reduced E_nodal_ across widespread regions compared with TD controls (Figure 4A). These regions included the triangular and orbital parts of the left inferior frontal gyrus (IFGtriang. L: *T* = −2.889, *p* = 0.005, *FDR-corrected*; ORBinf. L: *T* = −4.342, *p* < 0.001, *FDR-corrected*), the left insula (*T* = −2.996, *p* = 0.004, *FDR-corrected*), left amygdala (*T* = −2.813, *p* = 0.006, *FDR-corrected*), left supramarginal gyrus (*T* = −3.164, *p* = 0.002, *FDR-corrected*), and bilateral superior temporal poles (TPOsup. L: *T* = −4.455, *p* < 0.001, *FDR-corrected*; TPOsup. R: *T* = −2.754, *p* = 0.007, *FDR-corrected*). No significant differences in E_nodal_ were observed between adolescents with ASD and TD controls (Figure 4B). Ranking 28 social-brain regions by absolute E_nodal_ t-statistics revealed that TPOsup. L showed the largest group difference between children with ASD and TD controls. In contrast, the right insula showed the smallest absolute t-statistic (INS. R: *T* = −0.363*, p* = 0.718) (Supplementary Table 4). In addition, substantial inter-individual variability was observed in both groups, particularly in the left amygdala and bilateral superior temporal poles. Children with ASD exhibited broader distributions in these regions than TD controls.

## 4 Discussion

**Figure 5:**
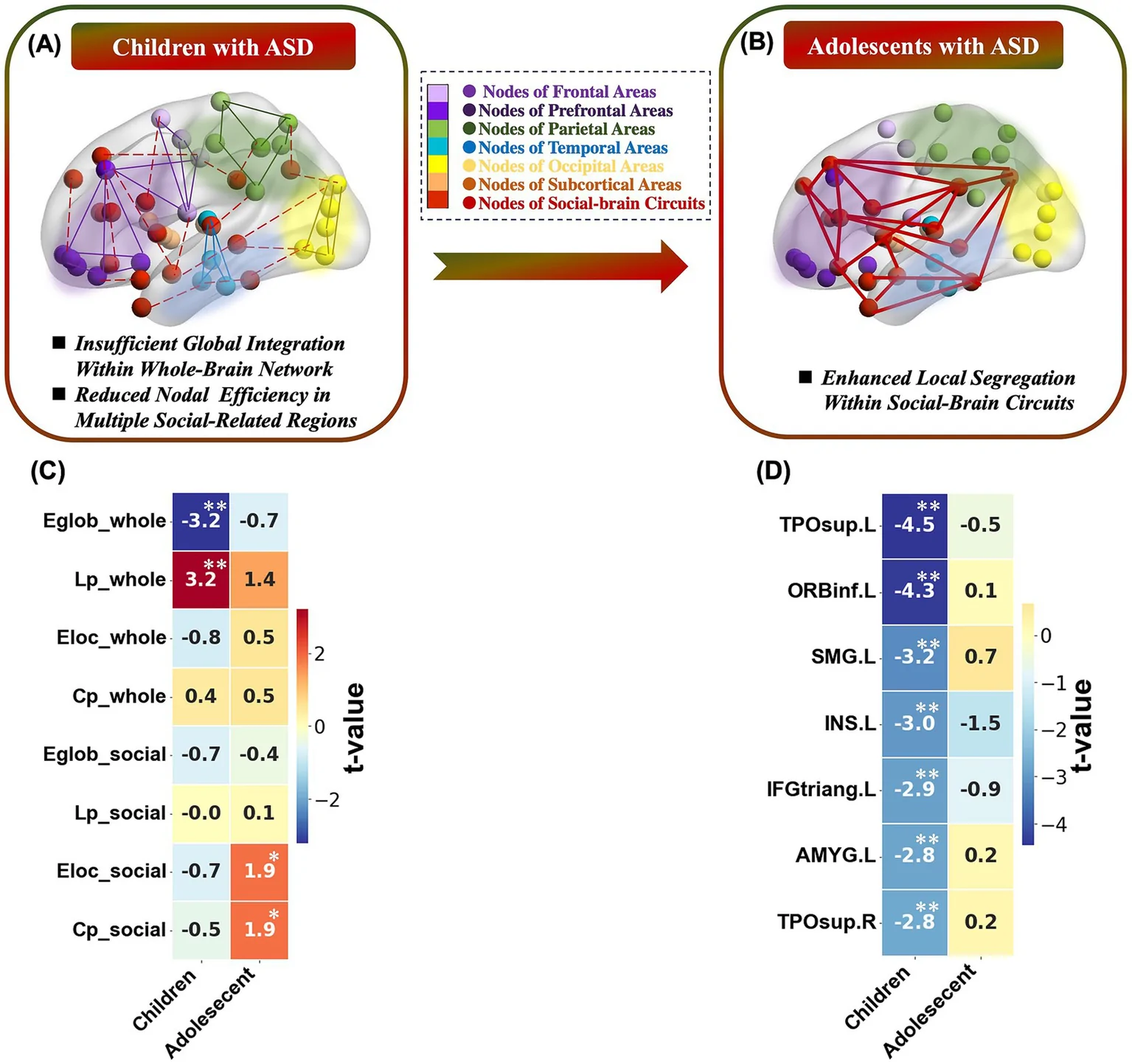
Summary of the dynamic development of atypical white matter structural networks in children and adolescents with ASD. **(A,B)** Nodes are color-coded according to their anatomical locations: Light purple for nodes of frontal areas; purple for nodes of prefrontal areas; green for nodes of parietal areas; blue for nodes of temporal areas; yellow for nodes of occipital areas; orange for nodes of subcortical areas. Social-related regions are highlighted in red nodes. The major cerebral lobes are shown in different colors: purple for the frontal lobe, green for the parietal lobe, blue for the temporal lobe, and yellow for the occipital lobe. Edges linking node pairs conceptualize the structural connections between cortical regions. Red broken lines represent weakened connections, whereas thick red lines indicate relatively strengthened connections. **(A)** Children with ASD showed reduced long-range connectivity (reduced global integration) across the whole-brain network and decreased nodal efficiency in several socially related regions. **(B)** Adolescents with ASD exhibited enhanced short-range connectivity (increased local segregation) within social-brain circuits. **(C,D)** Color-coded according to *t*-values of case–control comparisons: Red indicates positive *t*-values (ASD > TD), and blue indicates negative *t*-values (ASD < TD). The color bar represents the magnitude of the *t*-values. ** indicates a statistically significant group difference after FDR correction (*p* < 0.01). * indicates a statistically significant group difference before the multiple-comparison correction (*p* < 0.05, *uncorrected*). IFG, inferior frontal gyrus; ORBinf, orbital parts of inferior frontal gyrus; INS, insula; AMYG, amygdala; SMG, supramarginal gyrus; TPO, temporal poles; L, Left; R, Right. **(A)** Heatmaps showing the *t*-values of case–control comparisons for global network metrics in the child and adolescent groups. **(B)** Heatmaps showing the *t*-values for case–control comparisons of nodal efficiency in social brain regions in the child and adolescent groups.

In this study, we investigated alterations in the topological organization of WM structural networks in children and adolescents with ASD using DTI-based graph-theoretical analysis. First, cognitive assessment showed that although case–control differences in most intelligence indices tended to attenuate in the adolescent group, autistic symptoms remained prominent. At the global metrics level, young children with ASD exhibited disrupted WM topological organization, reflected by increased L_p_ and reduced E_glob_. In contrast, adolescents with ASD showed atypical topological properties within the social-brain subnetwork, characterized by increased C_p_ and E_loc_ relative to TD controls. Notably, adolescent-specific group differences in the social-brain subnetwork were sensitive to additional adjustment for FSIQ. Thus, the altered social brain network in adolescents with ASD may be partly influenced by intelligence ability. Nevertheless, the overall pattern of results still indicates that topological abnormalities in ASD differ between childhood and adolescence (Figure 5). In particular, strengthened short-range connections within social circuits during adolescence, together with other neurodevelopmental factors, may contribute to persistent autistic symptoms.

At the nodal metrics level, children with ASD showed reduced E_nodal_ in multiple social-related regions (Figure 5), including the left IFG (triangular and orbital parts), left insula, left amygdala, left SMG, and bilateral superior temporal poles. These results indicate that social brain circuits play a critical role in the neural mechanisms underlying autism. Our findings provide important insights into the neurobiological basis and developmental dynamics of ASD from the brain connectome perspective.

### 4.1 Atypical topological organization at the global level in ASD

The human brain can be conceptualized as a complex network supporting two complementary capacities for information processing: integration and segregation (Rubinov and Sporns, 2010). Integration reflects the coordinated communication across distributed brain regions, and is commonly quantified using L_p_ and E_glob_ (Farahani et al., 2019). L_p_ represents the average shortest-path length between all node pairs, with higher values indicating greater wiring costs for long-distance information transfer (Huang et al., 2015). Conversely, E_glob_ denotes the average inverse shortest-path length across all node pairs, with lower values reflecting reduced efficiency of global information communication (Latora and Marchiori, 2001). In the present study, young children with ASD exhibited increased L_p_ and decreased E_glob_, suggesting reduced network integration and insufficient long-range connectivity. Previous studies have also indicated that young children and adolescents with ASD exhibit elevated L_p_ or reduced E_glob_ in functional (Alaerts et al., 2015) and WM structural (Li et al., 2021) networks. These findings support the hypothesis that deficient long-distance connectivity is a core neurobiological feature of ASD (Just et al., 2007; Supekar et al., 2013). Insufficient long-range connectivity may disrupt interregional communication across the cortex, increasing wiring costs and decreasing the efficiency of integrating distributed information. No significant group differences in E_glob_ or L_p_ were observed in the adolescent group; these results aligned with previous findings suggesting that structural abnormalities in ASD tend to become less pronounced with development (Nunes et al., 2020; Karahanoğlu et al., 2018). One possible explanation is the atypical developmental trajectory in the autistic brain, which is characterized by early brain overgrowth, relatively slow growth during later childhood and adolescence, and more subtle structural alterations in adulthood (Courchesne et al., 2011). Consequently, the magnitude of case–control differences may gradually narrow during adolescence (Lange et al., 2015). In addition, adolescence represents an intermediate stage in the maturation of large-scale brain networks in typical development (Fair et al., 2007). Network integration measures change most prominently during childhood, whereas robust changes disappear in adolescence (Chen et al., 2013). Therefore, the atypical developmental pattern and puberty-specific characteristics may account for the absence of significant differences in the global network in adolescence. Future longitudinal studies with larger cohorts are needed to clarify the developmental trajectory of network integration across puberty in ASD.

Segregation reflects the capacity for specialized processing within local networks or short-range interconnections (Rubinov and Sporns, 2010). C_p_ and E_loc_ are commonly used for quantifying network segregation (Farahani et al., 2019). C_p_ measures the average proportion of a node’s neighbors that are interconnected; higher C_p_ indicates denser short-range clustering (Bullmore and Sporns, 2009). E_loc_ quantifies the efficiency of information exchange among a node’s immediate neighbors if the node is removed; higher E_loc_ reflects increased short-range connectivity or redundancy within local circuits (Latora and Marchiori, 2003). In the present study, the adolescents with ASD showed significantly higher C_p_ and E_loc_ within the social-brain subnetwork, indicating enhanced short-range connectivity and strengthened local interconnections within circuits relevant to social cognition. Increased local connectivity in WM structural networks in children with ASD has also been reported in previous studies (Li et al., 2014). These results align with the disrupted connectivity theory of autism, which posits that the autistic brain is characterized by long-range underconnectivity and short-range overconnectivity, contributing to the diverse cognitive and behavioral phenotypes of ASD (Belmonte et al., 2004; Wass, 2011). More specifically, Barttfeld et al. (2011) demonstrated that atypical functional networks in the autistic brain exhibit weakened long-range fronto-occipital connectivity coupled with enhanced short-range lateral-frontal connectivity. However, group differences in C_p_ and E_loc_ in adolescents were attenuated after additional adjustment for FSIQ. These findings indicate that the atypical segregation of the social-brain network in adolescents with ASD was sensitive to intelligence functioning. Compared with robust global network alterations in children, topological abnormalities in adolescents with ASD appear to be more susceptible to potential confounding factors. Therefore, the adolescence-specific reorganization of the social brain network in ASD requires further clarification in longitudinal cohort studies.”

Furthermore, the present findings may provide neurobiological support for the weak central coherence (WCC) theory, which proposes that individuals with ASD exhibit cognitive styles characterized by diminished global contextual integration and heightened attention to local details (Booth and Happé, 2018; Happé and Frith, 2006). The atypical brain-network organization observed in ASD, characterized by long-distance underconnectivity and local overconnectivity, is likely to serve as the neural basis for WCC cognitive profiles (Barttfeld et al., 2011; Just et al., 2004). However, the relationships between the global topological properties and autistic symptoms remain unclear. Although few studies (Li et al., 2021; Cai et al., 2022) have shown that altered global metrics are correlated with the ASD symptom severity, the findings are inconsistent and require further clarification. Moreover, individuals with ASD exhibit highly heterogeneous clinical presentations and brain-network characteristics (Guo et al., 2022). The pronounced inter-individual heterogeneity may partly account for the difficulty in clarifying specific relationships between global network topology and behavioral manifestations in ASD. Future studies that include objective behavioral assessments and multimodal MRI datasets, while accounting for the ASD heterogeneity, will be essential for elucidating the brain-behavioral relationships at the topological network level.

### 4.2 Altered topological organization of nodal properties in ASD

Regarding nodal attributes, we used E_nodal_ to evaluate the contributions of specific regions to information transmission across the network, with higher values indicating greater involvement in global communication (Feng et al., 2023). We observed significantly reduced E_nodal_ in the left IFG, left insula, left amygdala, left SMG, and bilateral superior temporal poles in young children with ASD. Similar alterations in these socially relevant regions have also been reported in previous network studies of ASD. For instance, reduced E_nodal_ in the left IFG has been observed in WM (Cai et al., 2022) and functional (Yoon et al., 2022) networks in ASD. In addition, disrupted nodal status of the left insula, amygdala, and SMG in ASD has been reported (Qian et al., 2021; Wang et al., 2023; Zhang et al., 2022). Lin et al. (2024) reported that individuals with ASD exhibit reduced E_nodal_ in bilateral superior temporal poles of the functional network compared to TD controls. These nodal alterations suggest diminished nodal contribution and impaired information-processing efficiency of these cortical regions in the autistic brain.

These altered regions are critical components of the neural systems implicated in ASD (Ecker, 2017). The left IFG, widely recognized as Broca’s area, plays an essential role in language processing (Skeide and Friederici, 2016) and non-verbal communication (Vandewouw et al., 2021). Dysfunction in the left IFG is linked to language communication deficits commonly observed in autism (Ecker et al., 2015). The insula and amygdala are core anatomical regions of the social–emotional brain network (Ecker, 2017). The amygdala plays a critical role in emotional regulation, whereas the insula contributes to self-, other-, and environmental awareness (Menon and Uddin, 2010). Moreover, the bilateral temporal poles are core regions for understanding others’ intentions and predicting social behavior (Olson et al., 2013). Abnormalities in the right temporal pole contribute to reduced social motivation and emotional expressivity (Rice et al., 2015). Considering these neurocognitive insights, we speculate that the impaired information-processing efficiency observed in these socially related regions may contribute to communication and social cognitive deficits in ASD. Notably, Cai et al. (2022) reported significant correlations between reduced E_nodal_ in the left IFG and the severity of autistic symptoms. In addition, Qian et al. (2021) found that altered nodal properties of the right amygdala are correlated with more severe ASD traits. However, these brain—behavioral relationships should be interpreted cautiously because complex social functions require cooperation among multiple brain regions, which are hardly localized to isolated areas. More specific experimental studies are needed to elucidate the elaborate correlations between nodal properties in social brain networks and social cognition deficits in ASD.

Furthermore, we found the TPOsup. L exhibited the largest group differences, and the INS. R showed the smallest absolute t-statistic. The superior temporal pole is considered a core hub of the social brain and plays essential roles in theory of mind, social semantic memory, and emotion integration (Olson et al., 2007). Deficits in the theory of mind are the most consistently reported cognitive impairments in children with ASD (Pelphrey et al., 2011). Therefore, the pronounced reduction in E_nodal_ of the TPOsup. L may provide additional neurobiological evidence for theory-of-mind dysfunction in ASD. The right insula is primarily involved in salience detection, attentional switching, and multisensory integration, serving as a key node of the salience network (Menon and Uddin, 2010). This regional pattern indicates that structural network alterations in ASD may not affect all components of the social brain equally. Regions supporting higher-order social cognition appeared to exhibit more pronounced topological alterations in the autistic brain.

Beyond the statistically significant group differences, violin plots revealed substantial inter-individual variability in E_nodal_ in both ASD and TD groups. This variability was particularly evident in the left amygdala and the bilateral superior temporal pole. The amygdala and bilateral superior temporal poles have been reported to undergo later maturation in structural connectivity and network organization, resulting in substantial individual variability (Saygin et al., 2015; Sun et al., 2024). Such developmental characteristics may contribute to the broad distribution of E_nodal_ observed in these regions. Furthermore, broader distributions in children with ASD suggest greater heterogeneity in the topological organization of the social brain network, consistent with the well-recognized clinical and neurobiological heterogeneity of ASD (Ecker et al., 2015; Guo et al., 2022). The heterogeneity of brain-network organization in ASD warrants further investigation in larger longitudinal studies.

### 4.3 Developmental changes in large-scale brain networks in ASD

Our results revealed that the topological disorganization of WM networks in ASD is characterized by insufficient global integration during childhood but enhanced local segregation during adolescence. These findings suggest that the disruptions in brain structural networks observed in individuals with ASD undergo dynamic changes across developmental stages. The inverse topological pattern observed between childhood and adolescence may result from atypical brain maturation and altered network reconfiguration processes in ASD. Large-scale brain networks typically show increasing integration and decreasing segregation from childhood to early adulthood (Fair et al., 2007; Feng et al., 2023; Hagmann et al., 2010). During this maturation process, long-range connections are strengthened and short-range connections are refined to optimize information processing with higher efficiency and lower wiring cost (Bullmore and Sporns, 2009; Fan et al., 2011). These developmental characteristics of topological organization are generally more prominent in structural networks than in functional networks (Cao et al., 2016). Our results revealed opposite developmental trends in ASD: diminished long-range connectivity in children, and excessive short-range connectivity in adolescents. This atypical trajectory aligns with findings from previous studies that suggest atypical developmental integration and segregation in WM networks as a potential neuropathological basis of autism (Fair et al., 2009).

Neural alterations during brain maturation likely contribute to the atypical topological organization observed in the autistic brain. Previous studies have demonstrated that the developmental trajectory of the structural networks is shaped by WM fiber maturation and synaptic pruning (Feng et al., 2023; Cao et al., 2014). Increased long-range connectivity is strongly associated with WM myelination, because more mature fibers and thicker myelin sheaths facilitate more efficient information transmission and reduced conduction cost (Fair et al., 2009). Short-range connections are regulated by synaptic formation and elimination, during which selective pruning of redundant synapses refines local network configuration (Huang et al., 2015; Collin and van den Heuvel, 2013). Progressive WM maturation and synaptic pruning collectively remodel the brain structural networks toward greater global integration and reduced local segregation. The disruptions in WM production, axonal myelination, and synaptogenesis observed in ASD may weaken long-range connectivity and enhance short-range connections. For example, emerging evidence indicates that myelination and oligodendrocyte generation are reduced in the autistic brain (Graciarena et al., 2018). Such hypomyelination and oligodendroglial deficits can impair neurotransmission and axonal conduction, thereby diminishing the efficiency of global information communication in ASD. In addition, insufficient elimination of redundant synaptic pathways has been demonstrated in animal models of ASD (Kim et al., 2017). These synaptic pruning abnormalities may lead to excessive connections within the local network. Moreover, previous studies on brain development have shown that WM maturation generally follows posterior-to-anterior, inferior-to-superior, and central-to-peripheral trajectories (Colby et al., 2011; Lebel et al., 2019). Furthermore, histological evidence indicates that myelination and synaptic elimination in frontal and temporal regions continue into young adulthood (Casey et al., 2005; Petanjek et al., 2011). These regions play critical roles in higher-order cognitive and socio-emotional functions. Their prolonged maturation reflects the ongoing reorganization of cortical neuronal circuitry. Such developmental processes may partly explain the more pronounced alterations in the segregation properties of social brain networks in adolescents with ASD. In addition, the longer developmental course of frontal-temporal regions may result in a less stable, more variable social brain network during childhood (Blakemore, 2008). These longer trajectories and greater individual variability may obscure group differences in the social brain network between children with ASD and TD controls.

Structural and functional brain alterations in ASD exhibit dynamic changes across developmental stages (Uddin et al., 2013; Courchesne et al., 2011). Several studies have indicated that structural brain abnormalities in ASD are prominent in childhood but tend to attenuate or approach normalization around puberty (Khundrakpam et al., 2017; Li et al., 2024; Mizuno et al., 2019). However, these neural alterations may not completely disappear; instead, they seem to manifest through other modalities or compensatory mechanisms. Atypical maturation is likely to exert persistent influences on the autistic brain, particularly on large-scale network organization. We hypothesize that these structural alterations are largely attributed to intrinsic neurodevelopmental processes. However, we cannot exclude that experience-dependent plasticity is also a key factor that influences brain-network organization, particularly during adolescence. Previous neurodevelopmental studies have reported that frequently engaged neural networks may become more strongly connected, whereas less frequently used networks may undergo less extensive WM maturation (Xin and Chan, 2020). Therefore, both intrinsic developmental processes and learning modulation are likely to contribute to brain-network organization in ASD. Disentangling their respective contributions will be important for clarifying neurobiological mechanisms of ASD and warrants further investigation in future longitudinal studies.

### 4.4 Strengths and limitations

The primary strength of this study is that it was conducted using relatively large multi-site DTI datasets, which enabled more robust insights than previous studies using smaller samples. In addition, we applied the TS harmonization method, a novel and effective approach to correcting measurement bias, to minimize site-specific effects arising from scanner and acquisition parameter differences. Furthermore, the present study extends prior research by examining topological disorganization in ASD from a developmental perspective, revealing dynamic alterations in large-scale brain networks between childhood and adolescence.

However, several unavoidable limitations should be acknowledged. First, we performed subgroup analyses to identify distinct patterns of topological disorganization between children and adolescents with ASD. However, the cross-sectional design of this study limited our ability to characterize longitudinal developmental changes within individuals. Therefore, findings across age groups should be regarded as age-related differences in topological alterations at the group level, rather than as evidence of individual developmental trajectories. Further longitudinal cohort studies are warranted to more precisely characterize dynamic network changes in the autistic brain at the individual level. Second, group differences in segregation measures within the social-brain subnetwork were weakened in the sensitivity analysis and did not survive FDR correction. These findings indicate that the topological alterations observed in adolescents should be interpreted with caution, because their robustness may be limited. Further validation in larger and better-characterized cohorts is needed to confirm these findings. Third, although deterministic tractography is widely used for reconstructing WM fiber bundles between pairs of brain regions, it has limited ability to resolve crossing-fiber configurations. More advanced approaches, such as probabilistic tractography, could be applied in future studies to address these issues. Finally, we used fiber count to define network edges, because it provides an intuitive estimate of macroscopic connectivity between brain regions. However, fiber count may not fully capture the complex microstructural properties of WM connections. Future studies employing multiple edge-weighting strategies are needed to provide a more comprehensive characterization of structural brain networks in ASD.

## 5 Conclusion

We investigated topological abnormalities and developmental variations in WM structural networks in ASD using DTI-based graph-theoretical analysis. Our results showed that individuals with ASD showed reduced global integration during childhood but showed heightened local segregation during adolescence. These age-related alterations align with the disrupted connectivity theory of autism and indicate atypical developmental modulations in the maturation and reconfiguration of WM structural networks in ASD. Additionally, we found that multiple social-related brain regions exhibited impaired information-processing efficiency, underscoring the critical involvement of social brain circuits in the neuropathology of ASD. Our findings provide robust evidence of atypical large-scale brain-network organization in ASD from the connectome perspective and highlight the importance of considering the developmental context when characterizing neural abnormalities in autism. However, future longitudinal and large-cohort studies that include multimodal neuroimaging and standardized clinical assessments are essential for elucidating how disruptions in brain-network development contribute to the manifestation and persistence of ASD symptoms.

## Data Availability

The raw data supporting the conclusions of this article will be made available by the authors, without undue reservation.

## Footnotes

1
https://www.nitrc.org/projects/mricrogl/

## Glossary

AAL: Automated Anatomical Labeling
ACC: Adjacent anterior cingulate cortex
ACG: Anterior cingulate and paracingulate gyri
AMYG: Amygdala
ANG: Angular gyrus
ASD: Autism spectrum disorder
AI: Anterior insula
CDM: Child developmental MRI project
Cp: Clustering coefficient
DICOM: Digital imaging and communications in medicine
DSM-5: Diagnostic and statistical manual of mental disorders, fifth edition
DTI: Diffusion tensor imaging
DWI: Diffusion-weighted images
Eglob: Global efficiency
Eloc: Local efficiency
Enodal: Nodal efficiency
EPI: Echo-planar imaging
FA: Fractional anisotropy
IFG: Inferior frontal gyrus
INS: Insula
FDR: False discovery rate
fMRI: Function magnetic resonance imaging
FSIQ: Full-scale intelligence quotient
Lp: Characteristic path length
MNI: Montreal neurological institute
mPFC: Medial prefrontal cortex
MRI: Magnetic resonance imaging
MTG: Middle temporal gyrus
NIfTI: The neuroimaging informatics technology initiative
ORBinf: Inferior frontal gyrus, orbital part
PRI: Perceptual reasoning index
PSI: Processing speed index
pSTS: Posterior superior temporal sulcus
ROI: Region of interest
SCI: Social communication and interaction
SFG: Superior frontal gyrus
SMG: Supramarginal gyrus
STG: Superior temporal gyrus
SRS-2: Social responsiveness scale, second edition
TD: Typically developing
TS: Traveling-subject
TPJ: Adjacent temporo-parietal junction
TPO: Temporal poles
VCI: Verbal comprehension index
WCC: Weak central coherence
WM: White matter
WMI: Working memory index
