# Supplementary material for: Atypical development of white matter structural networks in children and adolescents with autism spectrum disorder: a graph theory study
Source: Front Neurosci. 2026 Aug 25;20:1844698. doi: 10.3389/fnins.2026.1844698 (PMC13550125; doi:10.3389/fnins.2026.1844698)
Supplement: Supplementary file 1 [file Data_Sheet_1.docx]

Supplementary Material

# 1. Supplementary Figures and Tables

## 1.1 Supplementary Figures

**Supplementary Figure 1.** **Between-group comparisons of E_loc_/C_p_ within the whole-brain network.**

(A) Differences in the local efficiency of the whole-brain network between ASD and TD groups.
(B) Differences in the clustering coefficient of the whole-brain network between ASD and TD groups.

Light yellow represents children with ASD, light green denotes TD children, orange indicates adolescents with ASD, and cyan blue represents TD adolescents.

Statistical significance was set at *p* < 0.05. The comparative analysis revealed no statistically significant results in any group. ASD, Autism spectrum disorder; TD: Typically developing.

**Supplementary Figure 2.** **Between-group comparisons of L_p_/E_glob_ in the social brain subnetwork.**

(A) Differences in the characteristic path length of the social brain subnetwork between the ASD and TD groups
(B) Differences in the global efficiency of the social brain subnetwork between the ASD and TD groups.

Light yellow represents children with ASD, light green denotes TD children, orange indicates adolescents with ASD, and cyan blue represents TD adolescents.

Statistical significance was set at *p* < 0.05. The comparative analysis revealed no statistically significant results in any group.

ASD, Autism spectrum disorder; TD: Typically developing.

## 1.2 Supplementary Tables

**Supplementary Table 1. Site-specific scanning protocol**

| **Site** | ***Univ. of Osaka*** | | ***Univ. of Fukui*** | | ***Chiba Univ.*** | |
| --- | --- | --- | --- | --- | --- | --- |
| **No. of participants** | **18 ASD; 17 TD** | | **44 ASD; 41 TD** | | **7 ASD; 13 TD** | |
| **Scan parameters** | **T1** | **DTI** | **T1** | **DTI** | **T1** | **DTI** |
| Scanner | Signa Architect 3.0T | | Signa PET/MR 3.0T | | Discovery MR 750 3.0T | |
| Head coil | 48ch | | 8ch | | 32ch | |
| TR (ms) | 876.332 | 6000 | 8.488 | 9352 | 8.124 | 8500 |
| TE (ms) | 0.02 | 75 | 3.248 | 74.6 | 3.164 | 61.1 |
| FA (Def) | 5 | - | 11 | - | 15 | - |
| FOV (mm^2^) | 100 × 100 | 256 × 256 | 256 × 256 | 240 × 240 | 256 × 256 | 240 × 240 |
| Acquisition matrix | 256 × 256 | 128 × 128 | 256 × 256 | 128× 128 | 256 × 256 | 128 × 128 |
| Number of slices | 480 | 50 | 176 | 45 | 178 | 75 |
| Voxel dimension (mm^3^) | 0.94 × 0.94 × 0.50 | - | 1.0 × 1.0 × 1.0 | - | 1.0 × 1.0 × 1.0 | - |
| Slice thickness (mm) | 1 | 3 | 1 | 3 | 1 | 2 |
| Number of directions | - | 25 | - | 30 | - | 30 |
| *b* value (s) | - | 0/1000 | - | 0/1000 | - | 0/1000 |

TR, repetition time; TE, echo time; FA, flip angle; FOV, field of view; DTI, diffusion tensor imaging.

**Supplementary Table 2. Assessment of the homogeneity of variances for global and nodal topological metrics using Levene’s test.**

| **Topological Metrics** | | | ***Children Group*** | | | ***Adolescents Group*** | | | |
| --- | --- | --- | --- | --- | --- | --- | --- | --- | --- |
|  |  |  | ***F*** | | ***p-value*** | ***F*** | | ***p-value*** | |
| **Global Metrics** | **Whole-brain Network** | **E_glob_** | | 0.001 | 0.977 | 2.756 | 0.102 | |  |
|  |  | **L_p_** | | 1.854 | 0.178 | 1.726 | 0.194 | |  |
|  |  | **E_loc_** | | 0.731 | 0.395 | 0.107 | 0.745 | |  |
|  |  | **C_p_** | | 0.085 | 0.771 | 0.104 | 0.748 | |  |
|  | **Social-brain Subnetwork** | **E_glob_** | | 0.327 | 0.569 | 6.006 | 0.017 | |  |
|  |  | **L_p_** | | 0.170 | 0.682 | 3.916 | 0.052 | |  |
|  |  | **E_loc_** | | 1.567 | 0.215 | 6.826 | 0.017 | |  |
|  |  | **C_p_** | | 1.064 | 0.306 | 7.263 | 0.011 | |  |
| **Nodal Metrics** | **Nodal Efficiency** | **IFGtriang.L (13)** | | 0.367 | 0.547 | 0.152 | 0.698 | |  |
|  |  | **ORBinf.L (15)** | | 4.961 | 0.029 | 2.330 | 0.132 | |  |
|  |  | **INS.L (29)** | | 1.718 | 0.194 | 10.81 | 0.002 | |  |
|  |  | **AMYG.L (41)** | | 1.578 | 0.213 | 0.030 | 0.862 | |  |
|  |  | **SMG.L (63)** | | 0.239 | 0.626 | 5.087 | 0.028 | |  |
|  |  | **TPOsup.L (83)** | | 3.142 | 0.081 | 1.882 | 0.175 | |  |
|  |  | **TPOsup.R (84)** | | 0.005 | 0.942 | 0.904 | 0.345 | |  |

**Note*. *p* < 0.05 indicated Significant heterogeneity of variances. In the children’s group, no significant heterogeneity of variances was observed only for nodal efficiency in ORBinf.L. In the adolescent group, significant heterogeneity of variance was observed for three global metrics of social-brain subnetwork (global efficiency, local efficiency, and clustering coefficient), and for nodal efficiency in the INS.L and SMG.L.

* E_glob_, global efficiency; L_p_, characteristic path length; E_loc_, local efficiency; C_p_, clustering coefficient; IFG, inferior frontal gyrus; ORBinf, orbital parts of inferior frontal gyrus; INS, insula; AMYG, amygdala; SMG, supramarginal gyrus; TPO, temporal poles; L, Left; R, Right.

**Supplementary Table 3. Comparison of statistical outcomes obtained using Student's *t*-test and Welch's *t*-test for metrics with unequal variances.**

| **Age Group** | **Topological**  **Metrics** | | ***Statistical effects in Student’s t-test and Welch’s t-test*** | | | | | | |
| --- | --- | --- | --- | --- | --- | --- | --- | --- | --- |
|  |  |  | ***T***  ***(Student)*** | | ***p-value***  ***(Student)*** | | ***T***  ***(Welch)*** | | ***p-value***  ***(Welch)*** |
| **Adolescent Group** | **Social-brain Subnetwork level** | **E_glob_** | 0.215 | **0.831** | | 0.215 | | **0.831** | |
|  |  | **E_loc_** | 2.164 | **0.034** | | 2.164 | | **0.035** | |
|  |  | **C_p_** | 2.183 | **0.033** | | 2.183 | | **0.034** | |
|  | **Nodal level** | ***N_nodal_* of INS.L (29)** | -1.006 | **0.318** | | -1.006 | | **0.319** | |
|  |  | ***N_nodal_* of SMG.L (63)** | 1.365 | **0.177** | | 1.365 | | **0.178** | |
| **Children Group** | **Nodal level** | ***N_nodal_* of ORBinf.L (15)** | -4.208 | **<0.001** | | -4.147 | | **<0.001** | |

***For all metrics showing significant heterogeneity of variances, the statistical results (*t*-values and *p*-values) obtained using Student's *t*-test and Welch's *t*-test were highly consistent. No changes in statistical significance were observed between the two methods, indicating that the group comparisons were robust to violations of the homogeneity-of-variance assumption.

* E_glob_, global efficiency; E_loc_, local efficiency; C_p_, clustering coefficient; N_nodal_, nodal efficiency; INS, insula; SMG, supramarginal gyrus; ORBinf, orbital parts of inferior frontal gyrus; L, Left.

**Supplementary Table 4. Ranking for between-group comparisons of nodal efficiency across ROIs within the social brain.**

| **Regions of**  **Social-brain subnetwork** | ***Group Comparison of Nodal Efficiency in Children Group*** | |
| --- | --- | --- |
|  | ***T*** | ***p-value*** |
| **TPOsup.L (83)** | **-4.455** | **<0.001 ^**^** |
| ORBinf.L (15) | -4.342 | <0.001 ^**^ |
| SMG.L (63) | -3.164 | 0.002 ^**^ |
| INS.L (29) | -2.996 | 0.004 ^**^ |
| IFGtriang.L (13) | -2.889 | 0.005 ^**^ |
| AMYG.L (41) | -2.813 | 0.006 ^**^ |
| TPOsup.R (84) | -2.754 | 0.008 ^**^ |
| SMG.R (64) | -2.421 | 0.018 |
| MTG.R (86) | -2.402 | 0.019 |
| AMYG.R (42) | -2.380 | 0.020 |
| ANG.R (66) | -2.371 | 0.020 |
| TPOmid.R (88) | -2.275 | 0.026 |
| MTG.L (85) | -2.268 | 0.026 |
| ACG.L (31) | -2.137 | 0.036 |
| TPOmid.L (87) | -1.866 | 0.066 |
| IFGoperc.L (11) | -1.809 | 0.075 |
| STG.R (82) | -1.797 | 0.077 |
| ACG.R (32) | -1.788 | 0.078 |
| SFGdor.L (3) | -1.693 | 0.095 |
| SFGmed.R (24) | -1.502 | 0.138 |
| SFGmed.L (23) | -1.492 | 0.140 |
| ANG.L (65) | -1.474 | 0.145 |
| STG.L (81) | -1.458 | 0.149 |
| SFGdor.R (4) | -1.340 | 0.185 |
| ORBinf.R (16) | -1.167 | 0.247 |
| IFGoperc.R (12) | -0.570 | 0.570 |
| IFGtriang.R (14) | -0.565 | 0.574 |
| **INS.R (30)** | **-0.265** | 1.792  |

Regions were ranked according to the absolute *t*-values obtained from the between-group comparison of nodal efficiency. Statistical significance was defined as *p* < 0.05 after FDR correction. ** *p* < 0.01 with FDR correction.

The left superior temporal pole (TPOsup.L) exhibited the largest between-group difference (*T* = −4.455, *p* < 0.001, *FDR-corrected*), whereas the right insula (INS.R) showed the smallest absolute *t*-value (*T* = −0.363, *p* = 0.718) between children with ASD and TD controls.

**Supplementary Table 5. Between-group comparisons of nodal degree centrality in ROIs involved in social cognition.**

| **Regions of Social-brain subnetwork** | ***Children Group*** | | ***Adolescents Group*** | |
| --- | --- | --- | --- | --- |
|  | ***T*** | ***p-value*** | ***T*** | ***p-value*** |
| SFGdor.L (3) | -0.453 | 0.652 | 1.126 | 0.264 |
| SFGdor.R (4) | -0.299 | 0.766 | -0.515 | 0.609 |
| IFGoperc.L (11) | -0.668 | 0.507 | -1.084 | 0.283 |
| IFGoperc.R (12) | 0.944 | 0.349 | -1.849 | 0.069 |
| IFGtriang.L (13) | -1.726 | 0.089 | -1.197 | 0.236 |
| IFGtriang.R (14) | 1.713 | 0.091 | 0.213 | 0.832 |
| ORBinf.L (15) | -3.162 | 0.002 | -0.988 | 0.327 |
| ORBinf.R (16) | 0.520 | 0.604 | 0.341 | 0.734 |
| SFGmed.L (23) | -0.928 | 0.357 | -0.988 | 0.327 |
| SFGmed.R (24) | -0.427 | 0.671 | -0.042 | 0.967 |
| INS.L (29) | -0.785 | 0.435 | -1.014 | 0.314 |
| INS.R (30) | 1.472 | 0.145 | 0.296 | 0.768 |
| ACG.L (31) | -0.619 | 0.538 | -1.949 | 0.056 |
| ACG.R (32) | -1.260 | 0.212 | 0.113 | 0.910 |
| AMYG.L (41) | -1.530 | 0.131 | 0.546 | 0.587 |
| AMYG.R (42) | -2.570 | 0.012 | 0.225 | 0.823 |
| SMG.L (63) | -3.660 | 0.000 | 1.133 | 0.262 |
| SMG (64) | -1.295 | 0.200 | 1.121 | 0.267 |
| ANG.L (65) | 0.148 | 0.883 | 0.584 | 0.561 |
| ANG.R (66) | -0.952 | 0.344 | 0.879 | 0.383 |
| STG.L (81) | -0.399 | 0.691 | -0.660 | 0.511 |
| STG.R (82) | 0.468 | 0.641 | 2.102 | 0.040 |
| TPOsup.L (83) | -3.321 | 0.001 | -0.847 | 0.400 |
| TPOsup.R (84) | -2.487 | 0.015 | 0.104 | 0.918 |
| MTG.L (85) | -1.018 | 0.312 | 2.448 | 0.017 |
| MTG.R (86) | -1.396 | 0.167 | -0.261 | 0.795 |
| TPOmid.L (87) | -1.274 | 0.207 | 0.871 | 0.387 |
| TPOmid.R (88) | -0.465 | 0.643 | -0.843 | 0.403 |

*Statistical significance was defined as *p* < 0.05 after FDR correction. However, none of the group comparisons of nodal degree centrality remained statistically significant after FDR correction.

**Supplementary Table 6. Between-group comparisons of nodal betweenness centrality in ROIs involved in social cognition.**

| **Regions of Social-brain subnetwork** | ***Children Group*** | | ***Adolescents Group*** | |
| --- | --- | --- | --- | --- |
|  | ***T*** | ***p-value*** | ***T*** | ***p-value*** |
| SFGdor.L (3) | 1.586 | 0.117 | 0.015 | 0.988 |
| SFGdor.R (4) | 1.959 | 0.054 | 0.171 | 0.865 |
| IFGoperc.L (11) | 3.152 | 0.002 | -1.338 | 0.186 |
| IFGoperc.R (12) | 0.931 | 0.355 | -1.948 | 0.056 |
| IFGtriang.L (13) | 3.121 | 0.003 | -0.151 | 0.880 |
| IFGtriang.R (14) | 1.560 | 0.123 | 0.974 | 0.334 |
| ORBinf.L (15) | -0.399 | 0.691 | 0.610 | 0.544 |
| ORBinf.R (16) | 2.266 | 0.027 | 0.495 | 0.623 |
| SFGmed.L (23) | 1.201 | 0.234 | -0.381 | 0.704 |
| SFGmed.R (24) | 0.448 | 0.656 | 0.550 | 0.584 |
| INS.L (29) | 1.712 | 0.091 | 0.145 | 0.885 |
| INS.R (30) | 1.758 | 0.083 | -0.920 | 0.361 |
| ACG.L (31) | 1.773 | 0.081 | -1.633 | 0.108 |
| ACG.R (32) | -0.698 | 0.488 | 1.309 | 0.195 |
| AMYG.L (41) | -0.892 | 0.376 | -0.107 | 0.916 |
| AMYG.R (42) | -1.610 | 0.112 | 0.683 | 0.497 |
| SMG.L (63) | -0.237 | 0.814 | 2.102 | 0.040 |
| SMG (64) | 1.284 | 0.204 | -0.145 | 0.885 |
| ANG.L (65) | 0.546 | 0.587 | 0.355 | 0.724 |
| ANG.R (66) | -0.552 | 0.583 | 1.209 | 0.231 |
| STG.L (81) | 2.346 | 0.022 | -0.140 | 0.889 |
| STG.R (82) | 1.497 | 0.139 | 0.237 | 0.813 |
| TPOsup.L (83) | -3.746 | 0.000^**^ | -0.092 | 0.927 |
| TPOsup.R (84) | -1.413 | 0.162 | 0.758 | 0.451 |
| MTG.L (85) | 2.338 | 0.022 | 2.121 | 0.038 |
| MTG.R (86) | -0.100 | 0.921 | -0.011 | 0.991 |
| TPOmid.L (87) | 0.364 | 0.717 | 1.768 | 0.082 |
| TPOmid.R (88) | 0.718 | 0.475 | 0.230 | 0.819 |

*Statistical significance was defined as *p* < 0.05 after FDR correction.

After FDR correction, a significant between-group difference in nodal efficiency remained only in the left superior temporal pole (TPOsup.L) in the child group (*T* = −3.746, *p* < 0.001, *FDR-corrected*), with children with ASD showing reduced nodal betweenness centrality compared with TD controls. No significant between-group differences remained in any other social brain regions after FDR correction.
